# Long-Term Respiratory Muscle Endurance Training in Patients with Myasthenia Gravis: First Results after Four Months of Training

**DOI:** 10.4061/2011/808607

**Published:** 2011-07-07

**Authors:** Beate Rassler, Grit Marx, Stephanie Hallebach, Petra Kalischewski, Irene Baumann

**Affiliations:** ^1^Carl Ludwig Institute of Physiology, University of Leipzig, 04103 Leipzig, Germany; ^2^Neurological Joint Practice, 04229 Leipzig, Germany; ^3^Division of Neurology, Medical Care Center, Helios Hospitals, 04420 Markranstädt, Germany

## Abstract

Myasthenia gravis (MG) is characterized by reduced muscle endurance and is often accompanied by respiratory complications. Improvement of respiratory function is therefore an important objective in MG therapy. A previous study demonstrated that respiratory muscle endurance training (RMET) over four weeks increased respiratory muscle endurance of MG patients to about 200% of baseline. The purpose of the present study was to establish an appropriate maintenance training and to test its effects over four months. Ten patients with mild to moderate MG participated in this study. During the first month, they performed five training sessions per week. For the following 3 months, training frequency was reduced to five sessions per two weeks. Myasthenia score, lung function, and respiratory endurance were determined prior to training, after the first month, and after 4 months. Myasthenia score improved from 0.71 ± 0.1 to 0.56 ± 0.1 (*P* = 0.007). Respiratory endurance time increased from 6.1 ± 0.8 to 20.3 ± 3.0 min (*P* < 0.001). In conclusion, this RMET maintenance program is feasible and is significantly beneficial for MG patients.

## 1. Introduction

Myasthenia gravis (MG) is an autoimmune disease characterized by blockade of the neuromuscular synapse. Hence, muscle strength and, particularly, endurance are reduced, ensuing in increased muscular fatigue [1, 2]. In most MG patients, the entire muscular system is concerned, and this may also involve respiratory muscles. Despite normal spirometric values, patients with generalized MG often present a characteristic “myasthenic pattern” with decreasing respiratory volumes during MVV [3] and reduced respiratory muscle endurance [2]. Respiratory muscle dysfunction can further deteriorate patients' physical fitness and evoke upper airway obstruction [4], sleep apnea [5, 6], or even respiratory failure as the characteristic feature of myasthenic crisis [7, 8]. Improvement of respiratory muscle function is therefore an important objective in MG therapy. 

In addition to pharmacological or operative treatment, exercise therapy can be used as an adjuvant method in therapy of MG [9, 10]. Besides general exercise programs, specific respiratory muscle training could be beneficial especially for patients with compromised respiratory function. Positive effects of respiratory muscle training on respiratory muscle strength and endurance in patients with pulmonary disorders were demonstrated for the first time by Keens et al. [11]. Likewise, in patients with neuromuscular diseases respiratory dysfunction due to inadequate function of respiratory muscles is a strong rationale for a specific training of respiratory muscles. Numerous studies on respiratory muscle training have been performed in patients with spinal cord injury [12, 13], postpolio syndrome [14], or with neuromuscular disorders such as Duchenne's muscular dystrophy or spinal muscular atrophy [15, 16] demonstrating improvement of lung function and of respiratory muscle strength and/or endurance. 

In contrast, there is only little experience with specific respiratory training in MG patients [18–20]. These studies reported beneficial effects of respiratory training on respiratory muscle strength and/or lung function. None of these studies applied sustained hyperpnea for training. However, maintenance of an elevated level of ventilation over a longer period of time such as in situations of increased physical activity would be an appropriate training for patients with increased respiratory muscle fatigue. 

Several years ago, we used a normocapnic hyperpnea training that had been previously applied in healthy untrained and trained subjects [21–23] as well as in patients with chronic obstructive pulmonary disease (COPD) [24, 25]. This type of respiratory muscle endurance training (RMET) has also been applied in MG patients in a previous study [26]. Four weeks of this normocapnic hyperpnea training in MG patients resulted in a more than twofold enhancement of respiratory muscle endurance as reflected by time to exhaustion (*T*_Lim_) and total ventilated volume (*V*_Lim_) in a respiratory endurance (RE) test. However, this gain in respiratory muscle endurance reduced after termination of the training period. Maintaining improved respiratory muscle endurance requires to regularly continue RMET. This might be hampered by strenuousness and expenditure of time associated with the training. Therefore, the aim of the present study was to establish a maintenance training program and to test it for feasibility and benefit with respect to respiratory muscle endurance, MG symptoms, and lung function. 

## 2. Methods

### 2.1. Subjects

The patients involved in this study were regularly consulting two neurologists specialized in MG who were involved in this study (IB, PK). We chose 27 patients with mild to moderate generalized MG (degree II according to MGFA classification [17], degree 1–3 according to Oosterhuis classification [1]) as possible participants. They have been suffering from MG for 1–39 years. Patients with ocular symptoms only and hospitalized patients were excluded. Eleven of the preselected patients resigned from participation in the training study due to problems with transportation or with their time schedule (5 patients), or because they felt no need of respiratory training (6 patients). Six patients tried the use of the training device but did not manage the rebreathing technique, the use of the training device, and the training frequency. In the end, 10 patients (5 male, 5 female, average age 60 ± 4.2 y) participated in the study. Characteristics of these patients are given in Table 1. Five of them had already participated in our first RMET study several years ago [26]. All participants had experienced respiratory symptoms or problems in the past due to their myasthenia and, for this reason, were motivated to perform the respiratory endurance training. Seven patients had additional chronic diseases; five suffered from arterial hypertension, two of them additionally from diabetes mellitus. One patient had a coronary heart disease, and one other patient had a Lupus erythematodes. All patients were free from chronic respiratory diseases. None of the patients smoked at present. Two patients were ex-smokers but had ceased smoking at least fifteen years before. All participating patients gave their written informed consent. The study was approved by the local ethics committee. 

### 2.2. Study Protocol

The study consisted of two phases. Phase 1 included a four-week training period; during phase 2, the training was continued for another three months. The protocol of phase 1 was the same as applied in the first study [26] but without a detraining period. In brief, all patients including those who had already participated in the previous study received a detailed explanation and demonstration of all testing and training details and then practiced the use of the training device at home for one week 10 min per day. The pretraining tests (baseline, B) were performed 6–8 weeks later. They contained an MG score (Besinger score [17, 27]), lung function testing, and an RE test. For lung function tests including spirometry and maximal voluntary ventilation (MVV) and for the RE test, we used a metabolic cart (MetaMax 3B, Cortex Biophysik GmbH, Leipzig, Germany). Additionally, respiratory muscle strength (maximum inspiratory pressure, PI_max_) at residual volume (RV) was determined (resPI_max_, Andos, Hamburg, Germany). For the RE test, patients connected their training device to the metabolic cart and breathed with a tidal volume (*V*_*T*_) ranging between 50 and 75% of VC at a rate of 25–40 breaths per minute at normocapnic conditions. This set-up was intended to induce test termination after a maximum of 10–12 min. Criteria to terminate the test were patients' perception of exhaustion or reduction in ventilation (${\dot{V}}_{E}$) by more than 10% of the target for 1 min. This test was accomplished at least two times on separate days with the best test being evaluated. We measured endurance time (*T*_Lim_: time until test termination) and endurance volume (*V*_Lim_: total volume breathed during the test, calculated as *T*_Lim_ multiplied by ${\dot{V}}_{E}$).

The normocapnic hyperpnea training started after completion of the baseline tests. During phase 1, all patients accomplished 20 training sessions in a period of 4–6 weeks with about five training days and two resting days per week. Each training session lasted 30 min. Patients achieved isocapnia by using a portable rebreathing device as described in detail by Markov et al. [23], thus performing partial rebreathing. Patients were carefully coached beforehand to be attentive to sensations of air hunger or dizziness as symptoms of hypercapnia or hypocapnia. Additionally, we repeatedly performed pCO_2_ measurements in the laboratory to assure normocapnia. Target values of ${\dot{V}}_{E}$, *V*_*T*_, and breathing rate (*f*_*R*_) were defined in the same range as in the previous study [26], that is, ${\dot{V}}_{E}$: 50–60% of individual MVV, *V*_*T*_: 50–60% of VC, *f*_*R*_: 25–35 min^−1^. Patients were instructed to perform the training at home always at the same time of a day and at a constant time interval after medication. After each training session, they had to fill in a short questionnaire regarding changes in MG symptoms (see Appendix) and to assess occurrence and degree of air hunger and respiratory effort using visual analogue scales. Moreover, after each training session they documented date, time of day, duration of session, estimated breath volume, pacing frequency, and, if necessary, problems or remarks.

During training phase 1, patients came at least two times to the laboratory and performed a training session with the device connected to the metabolic cart to assure correct performance and normocapnia during training. Additionally, we contacted all patients twice a week to ask for problems with the training or with their MG symptoms.

At least 10 days after completion of the last training session, a posttraining test series (P1) was carried out in the same way as the baseline test series. All examinations were performed at the same time of day as the pretraining tests. For RE test, ${\dot{V}}_{E}$, *V*_*T*_, and *f*_*R*_ were set to the same values as in the baseline test.

Training phase 2 started after conclusion of all P1 tests. In phase 2, training frequency was reduced to 5 training sessions per two weeks. Training settings were the same as in phase 1. One training session per month was performed in the laboratory to check correctness of training performance. Patients were phoned once per week to ask for possible problems, for MG symptoms, and for their subjective experience with training. At the end of phase 2, patients performed another posttraining test series (P4) identical to B and P1 tests. The patients were then asked to continue the training, and all participants agreed. 

### 2.3. Data Analysis

All values are given as mean values ± SEM. We evaluated Besinger score, vital capacity (VC), forced expiratory volume in 1 s (FEV_1_), peak expiratory flow (PEF), MVV, PI_max_, *T*_Lim_, and *V*_Lim_. Comparisons between the three test periods (B, P1, P4) were performed using a repeated measures analysis of variance with posthoc multiple comparisons according to the Holm-Sidak method. Additionally, when a significant difference was detected, a multiple linear regression was performed to describe the relationship of this respective variable with time (days since baseline tests) and with training (cumulated volume breathed during training). 

## 3. Results

### 3.1. Training Course

All patients completed at least 50 training sessions with 30 min training time per session as required. No complications were reported during the total observation time. In 8 patients, MG was stable throughout the time without change in medication or outpatient care by their neurologist. One patient (no. 7) got a respiratory infection at the beginning of phase 2 and received additional cholinesterase inhibitors for 4 weeks. In one other patient (no. 10) MG symptoms had slightly deteriorated 5 weeks prior to the training period so that he transiently needed a higher dose of cholinesterase inhibitors. During training, his symptoms gradually improved, and his medication could be adequately reduced. 

### 3.2. Besinger Score of MG Symptoms

**Figure 1:**
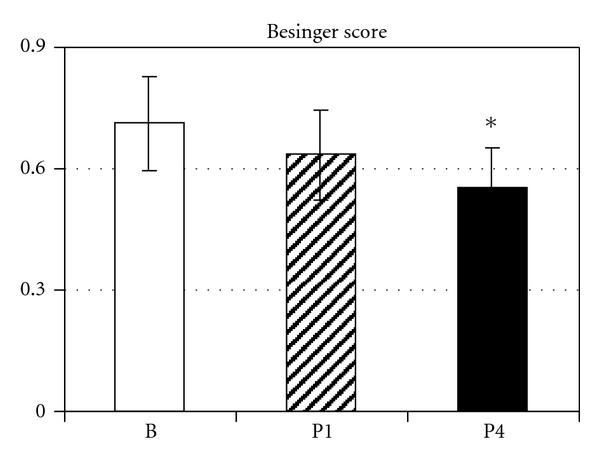
Besinger score of myasthenic symptoms before (B) and after 4 weeks (P1) and 4 months (P4) of respiratory endurance training. Data are presented as mean ± SEM; *significant difference versus B.

Besinger score ranges from 0 to 3 with 0 meaning the best value, that is, no myasthenic symptoms, and 3 meaning most severe symptoms [17, 27]. The participants of the study achieved a baseline Besinger score of 0.71 ± 0.12. Training significantly improved the score (*P* = 0.007). After phase 1 training period, the improvement was not significant (0.63 ± 0.11, *P* = 0.09), but after phase 2, we found a significant score reduction to 0.56 ± 0.10 (*P* = 0.002, Figure 1). A multiple linear regression showed no significant correlation with training (*P* = 0.09) or with time (*P* = 0.19). No deterioration in myasthenia symptoms related to respiratory training was reported in the training questionnaire. 

### 3.3. Lung Function

Baseline lung function was normal for all patients with VC being 95.5 ± 3.7%, FEV_1_ 90.5 ± 3.6%, PEF 86.9 ± 4.5%, MVV 93.8 ± 6.6%, and maximal inspiratory pressure (PI_max_) 75.1 ± 5.4% predicted. RMET induced mild but not significant increases in VC (P1: 95.9 ± 3.5%, P4: 99.2 ± 4.1%, *P* = 0.39), FEV_1_ (P1: 93.1 ± 2.7%, P4: 96.3 ± 3.8%, *P* = 0.28), PEF (P1: 90.0 ± 4.8%, P4: 95.7 ± 5.4%, *P* = 0.07), MVV (P1: 95.6 ± 6.0%, P4: 101.0 ± 6.5%, *P* = 0.24), and PI_max_ (P1: 79.2 ± 5.4%, P4: 78.0 ± 6.4%, *P* = 0.42). Absolute values are given in Table 2. 

### 3.4. Respiratory Endurance Tests

**Figure 2:**
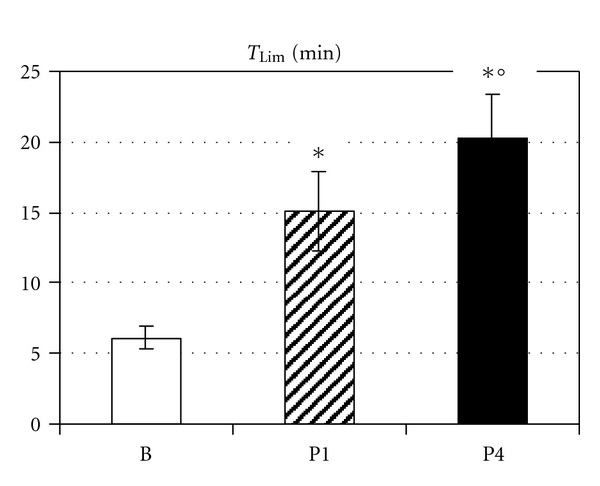
Respiratory endurance time (*T*_Lim_ (min)) before (B) and after 4 weeks (P1) and 4 months (P4) of respiratory endurance training. Data are presented as mean ± SEM; *significant difference versus B, °significant difference between P1 and P4.

**Figure 3:**
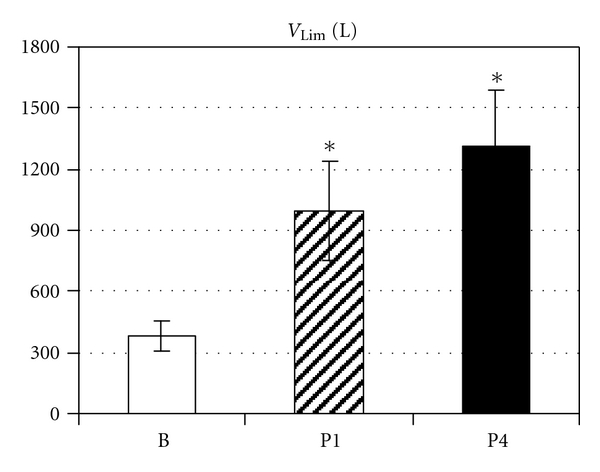
Total volume ventilated during respiratory endurance test (*V*_Lim_ (L)) before (B) and after 4 weeks (P1) and 4 months (P4) of respiratory endurance training. Data are presented as mean ± SEM; *significant difference versus B.

In the baseline RE test, patients achieved an average time to exhaustion (*T*_Lim_) of 6.1 ± 0.8 min at an average ventilation of 58.9 ± 4.7 L min^−1^ corresponding to 54.7 ± 2.5% MVV. The patients breathed during *T*_Lim_ a total volume (*V*_Lim_) of 382 ± 78 L. RMET significantly increased *T*_Lim_ and *V*_Lim_ (*P* < 0.001). After four weeks, *T*_Lim_ reached 15.1 ± 2.8 min and *V*_Lim_ 995 ± 243 L. Prolonged training further increased *T*_Lim_ to 20.3 ± 3.0 min (Figure 2) and *V*_Lim_ to 1316 ± 275 L (Figure 3). A multiple linear regression showed significant correlation with training and with time (*P* < 0.001) for *V*_Lim_. *T*_Lim_ was significantly correlated with training (*P* = 0.005) but not with time (*P* = 0.10). 

## 4. Discussion 

In the present study, we established and evaluated a home-based RMET program appropriate for long-term application in patients with mild to moderate myasthenia gravis. A tight control consisting of a careful training documentation and self-reported questionnaires, frequent phone calls, and regular laboratory tests ensured adequate training performance. The results demonstrated that 30 min normocapnic hyperpnea training 2-3 times per week over 3 months induced further improvement of respiratory muscle endurance additionally to the gain achieved after a 4-week intensive training period (phase 1). 

The results of phase 1 confirm our previous results obtained with the same training program, that is, 30 min normocapnic hyperpnea training 5 times per week over 4 weeks [26]. Most of the participants of the previous study felt the number of training sessions per week being too high to perform the RMET program regularly over a long time. The reduced training frequency applied in phase 2 was acceptable for all of the participants; therefore, all agreed to continue the RE training for at least 3 further months.

We had expected the training program in phase 2 to maintain respiratory endurance expressed by *T*_Lim_ and *V*_Lim_ at the enhanced level achieved after phase 1 (about 250% of baseline). However, even with the lower training frequency, *T*_Lim_ and *V*_Lim_ further increased during the following 3 months to about 340% of baseline. 

Moreover, myasthenia symptoms indicated by Besinger score improved significantly compared to baseline. After four weeks of training (phase 1), we only observed a tendency to improvement, thus confirming results of our previous study [26]. During phase 2, enhancement of Besinger score progressed and reached significance after 4 months of RMET. Correspondingly, patients reported subjective improvement of their general state, reduced exhaustion in many activities of daily life, and attenuation of myasthenia symptoms. Recent reviews on exercise therapy, especially respiratory muscle training, in neuromuscular disease demonstrated limited positive effects of training therapy on pulmonary rehabilitation, exercise tolerance, and quality of life [28, 29]. 

RMET improved lung function (VC, FEV_1_, PEF, MVV, PI_max_) of MG patients slightly but not significantly. Lung function parameters such as VC, FEV_1_, PEF, and PI_max_ are based on short maneuvers requiring maximal effort. These abilities are usually not reduced in patients with mild to moderate MG. All of our patients had normal lung function at baseline reflecting a moderate degree of respiratory muscle weakness. A significant reduction of total lung capacity and hence, of VC can be expected when inspiratory muscle force is reduced by about 50% [30]. In healthy subjects, respiratory endurance training had no effect on lung function [21, 22]. Training effects also depend on duration and intensity of training. This is expressed by significant correlation of *T*_Lim_ and *V*_Lim_ with cumulated volume breathed during total training time. In MG patients, respiratory muscle training at 6 days per week over 3 months significantly improved static lung volumes such as VC and FEV_1 _[18]. Accordingly, we observed further increase in these volumes during phase 2 of RMET in our patients. An 8-week inspiratory muscle training performed three times per week did not significantly change FVC and FEV_1_ but significantly improved MVV and PI_max_ [20]. The increase in MVV was 8% in their study which was in a similar range as in our study. 

The different effects of respiratory muscle training on lung function might also be explained by the training specificity of respiratory muscle training as already described by Leith and Bradley [31] who showed that respiratory strength training mainly improved maximal force. This is reflected in the two studies mentioned above [18, 20]. Their training programs were predominantly directed on respiratory muscle strength training, and both groups found a significant increase in PI_max_. On the contrary, respiratory endurance training predominantly improves endurance which may be accompanied by a mild positive effect on force. Improved respiratory endurance is even more important than improvement of lung function parameters in MG patients. Weakness and fatigue of respiratory muscles is responsible for dyspnea and reduced exercise tolerance and thus, can compromise quality of life and increase the risk of respiratory failure [32]. In healthy subjects, RMET reduced respiratory muscle fatigue and increased cycling endurance in those subjects who had presented more than 10% of diaphragm or abdominal muscle fatigue in a pretraining exhaustion test [33]. As increased muscular fatigue is a characteristic feature of MG, a similar outcome had been expected for MG patients and was reflected in enhanced *T*_Lim_ and *V*_Lim_. Moreover, all our patients perceived benefit of the training in terms of improved respiration and relief of respiratory symptoms. None of them reported any adverse effects. This is reflected best in the fact that all participants agreed to continue the training study.

**Limitations of the Study**

The main limitation of this study is the lack of a control group. The study program was strenuous and, particularly in phase 1, time consuming. Even the control program would have needed much time and effort as control patients also had to complete all laboratory tests. Hence, those patients who had resigned from training also refused to serve as controls. Participation in the RE training study required high motivation. Six patients who were asked for participation in this study refused as they did not see a necessity to perform this training. On the other hand, patients who had respiratory symptoms or had experienced respiratory disturbances in their past were highly motivated to perform the respiratory endurance training but were not willing to serve as nontraining controls. For these reasons, no control group could be formed.

Moreover, RMET cannot be applied to all MG patients. Some patients may not cope with the technique, and patients with severe MG are not able to perform this training at sufficient intensity. For patients with mild to moderate MG, this normocapnic hyperpnea training is appropriate if patients are motivated to learn the technique and to subject to the time need and effort of the training. These patients can considerably improve their respiratory muscle endurance.

A long-term endurance training program is expected to improve muscle endurance by inducing muscular hypertrophy. In the present study, we could not clarify whether reduced perception of respiratory effort rather than true respiratory muscle hypertrophy caused the improvements observed in the study. A recent reevaluation of 15 years of RMET experience in healthy subjects revealed that enhanced muscle endurance after RMET was unlikely due to reduced adverse respiratory sensations [34]. However, this does not preclude other factors such as improved neuromuscular coordination that may contribute to the RMET effect.

In conclusion, the study demonstrated that respiratory endurance training can be performed safely in patients with mild or moderate MG over several months. It indicates that this training program could be appropriate for long-term, ideally life-long, application resulting in improvement of respiratory muscle endurance and myasthenia symptoms.

## Figures and Tables

**Table 1:** Characterization of patients participating in respiratory endurance training.

Patient	Gender	Age (y)	BMI (kg/cm^2^)	MG degree		Diagn. (y)	Medication	
				MGFA	OO		ChEI (mg/d)	IT (mg/d)
1	F	43	29.3	IIa	2	2	180	
2	M	62	27.4	IIa	2	9	420	100
3	F	33	19.4	IIb	3	11	240	
4	M	66	32.4	IIa	2	9		100
5	M	75	25.9	IIa	2	4	300	
6	F	54	23.9	IIa	2	15	105	
7	F	63	30.7	IIa	2	39	240	
8	M	68	26.4	IIa	1	2		150
9	F	73	26.6	IIa	2	2	420	
10	M	67	30.5	IIa	2	2	310	

M: male; F: female; BMI: body mass index; MG degree: degree of myasthenia gravis according to classifications of MGFA (Myasthenia Gravis Foundation of America [17]) and OO (Oosterhuis [1]); Diagn.: years since MG diagnosis; ChEI: cholinesterase inhibitors; IT: immunotherapy with azathioprine.

**Table 2:** Lung function data.

Patient	VC (L)			FEV_1_ (L)			PEF (L s^−1^)			MVV (L min^−1^)			PI_max_ (kPa)		
	B	P1	P4	B	P1	P4	B	P1	P4	B	P1	P4	B	P1	P4
1	3.8	4.1	4.2	3.1	3.3	3.9	7.1	6.2	7.6	142	150	133	9.1	9.4	9.5
2	3.9	3.8	4.0	3.1	3.1	3.1	8.6	8.0	8.4	138	131	188	9.5	10.9	11.4
3	3.1	3.4	3.3	2.3	2.6	2.4	4.5	5.1	4.7	74	73	84	5.6	6.9	7.0
4	3.7	3.7	3.6	2.9	3.2	2.9	7.6	9.4	9.0	130	152	144	8.9	8.9	8.3
5	3.7	3.4	3.5	2.8	2.5	2.5	7.7	7.8	8.9	124	121	114	4.3	5.3	5.3
6	3.9	3.6	4.4	2.7	2.6	3.5	4.7	5.3	7.2	73	89	105	6.6	5.8	6.3
7	2.8	2.7	2.6	2.1	2.1	2.0	4.9	5.1	4.4	91	91	97	5.6	6.6	5.1
8	4.6	4.7	4.4	3.1	2.9	2.9	7.0	7.4	7.4	162	154	155	8.1	9.0	9.3
9	2.6	2.6	2.8	2.1	2.4	2.3	4.6	4.4	5.1	76	75	81	5.9	5.1	4.6
10	2.7	2.9	3.5	1.9	2.2	2.5	7.1	7.6	7.5	94	92	90	5.9	5.8	6.1

Mean	**3.5**	**3.5**	**3.6**	**2.6**	**2.7**	**2.8**	**6.4**	**6.6**	**7.0**	**110**	**113**	**119**	**6.7**	**7.1**	**7.1**
SEM	0.20	0.20	0.20	0.15	0.13	0.18	0.49	0.52	0.53	10.3	10.3	11.1	0.57	0.67	0.75

VC; vital capacity; FEV_1_; forced expiratory volume in 1 s; PEF; peak expiratory flow; MVV; maximal voluntary ventilation; PI_max _; maximal inspiratory pressure. B; baseline values; P1; posttraining test after 4 weeks of training (phase 1); P4; posttraining test after 4 months of training (phase 2).

## Appendix

### Self-Reported Training Questionnaire

The original questionnaire as presented to the patients was in German.

Training session no.: …   Date: …
Have symptoms of your myasthenia changed after this training?
  Yes
  No
If yes, which symptoms/functions have deteriorated?
  ○ Ptosis
  ○ Double vision
  ○ Swallowing problems/dysphagia
  ○ Chewing problems
  ○ Pursing lips/whistling
  ○ Neck strength (e.g., difficulties with holding your head up?)
  ○ Arm strength (e.g., problems with hair-drying?)
  ○ Leg strength (e.g., difficulties with climbing a staircase?)

How long did this deterioration continue? …
Was additional medication (cholinesterase inhibitors) necessary?
  Yes
  No
Did you have to interrupt or break off this training due to deterioration of MG symptoms?
  Yes
  No
Further remarks concerning your current state: …
